# Computerised tomography findings in HIV-associated cryptococcal meningoencephalitis at a tertiary hospital in Pretoria

**DOI:** 10.4102/sajr.v21i2.1215

**Published:** 2017-11-14

**Authors:** Nausheen Khan, Juliane Hiesgen

**Affiliations:** 1 Kalafong Hospital, University of Pretoria, South Africa

## Abstract

**Objective:**

Computerised tomography (CT) scans of 30 patients, admitted with HIV-associated cryptococcal meningoencephalitis (CM), were retrospectively reviewed and the different neuroradiological findings categorised. In addition to the characterisation of the cohort, we looked at whether positive CT scans can indicate negative outcomes when compared with normal imaging.

**Methods:**

We identified all patients admitted with CM to Kalafong Hospital in Pretoria, South Africa, over a 2-year period and selected those who underwent brain CT. Abnormal findings were divided into cryptococcal-related lesions and other pathological findings. Clinical data, as well as laboratory data and information about the outcomes were collected.

**Results:**

Thirty-nine (44.8%) out of 87 patients had a CT done during the hospital admission, of which 30 were reviewed and independently reported by the authors. The majority of CT scans were non-contrasted (*n* = 21). Four patients (13.3%) had normal imaging. Amongst the 26 patients with abnormal CTs, we found 16 brain scans (53.3%) with changes most likely attributed to CM. Dilated Virchow–Robin (VR) spaces, found on eight scans (26.7%), were the most common CT finding related to neurocryptococcosis. Global cerebral atrophy, present in 17 patients (56.7%), was the prevailing generalised abnormality. The mortality of all patients who underwent imaging was similar (33.3%) to the mortality in the total cohort of patients with cryptococcal meningitis (31%). In the group with cryptococcal-related changes on imaging, the mortality was higher (53.3%) than in both groups and a subgroup of five patients with hydrocephalus showed 100% mortality.

**Conclusion:**

Computerised tomography brain imaging was performed in 44.8% of all patients admitted with CM into our hospital. More than half of the images showed cryptococcal-related pathological findings, of which dilated VR spaces were the most common. Only 13.3% of scans were normal. Mortality was higher in the patients with cryptococcal-related pathology (53.3% vs. 31%), with hydrocephalus being associated with a 100% mortality. No scan in our cohort showed any pathology requiring neurosurgical intervention or contraindicating the procedure of a lumbar puncture.

## Introduction

Cryptococcal meningitis is the most common life threatening fungal infection in people living with HIV/AIDS^1^ and the second most common opportunistic infection after tuberculosis (TB) in South Africa.^2^ Prevalence and mortality in South Africa are high,^3^ and the long-term prognosis is very poor.^4^ This is despite the good availability of antiretroviral (ARV) therapy and national treatment guidelines for neurocryptococcosis.

Brain imaging in patients with AIDS and central nervous system (CNS) involvement is important, not only for assessing the severity of the presenting entity [e.g. cryptococcal meningoencephalitis (CM)] but also for looking at the degree of underlying neurodegeneration (HIV-related) and to diagnose concomitant conditions (e.g. additional vascular disease, other infections such as neurocysticercosis or TB, tumours, etc.). Most authors, therefore, distinguish between cryptococcosis-related and ‘other’ lesions.^5,6^

In a previous study from Kalafong Hospital, Pretoria, we looked at the clinical presentation and outcomes of all patients admitted with cryptococcal meningitis over a 2-year period.^7^ However, because of the high burden of patients and the limited resources, only a relatively small number of admitted patients with cryptococcal meningitis underwent cerebral imaging so that we did not analyse the radiological data.

The present study complements our previous findings by reviewing the brain computed tomography (CT) scans available of HIV patients with cryptococcal meningitis seen in our institution from 2012 until 2013. We retrospectively analysed and categorised the neuroradiological findings and compared our results with other studies on imaging in neurocryptococcosis. In addition, we looked at outcomes in relevant subgroups of our cohort.

## Methods

All patients admitted with cryptococcal meningitis between January 2012 and December 2013 were retrospectively identified, via screening of the discharge summaries, and their files retrieved. Only confirmed cases [positive cerebrospinal fluid (CSF) diagnosis by India Ink Stain, Cryptococcal Antigen test and/or positive fungal culture] were included. In this spin-off study, we retrieved the CT scans of patients who underwent brain imaging during this period. All scans were acquired on the same Phillips brilliance 16 slice CT scanner, and the scans were assessed independently by a radiologist and a neurologist. No patient underwent Magnetic resonance imaging (MRI). Data including HIV- and treatment history, presenting symptoms, blood and CSF findings, complications and clinical outcomes (survival) were collected and analysed.

## Ethical consideration

Ethical approval was obtained by the Research Ethics Committee of the Faculty of Health Sciences, University of Pretoria (430/2013).

## Radiological findings

For better comparison with other published data, we attempted to categorise the CT abnormalities. Although no reported radiological changes in cryptococcal meningitis seem to be specific or unique for the infection, some findings are common and thought to be cryptococcal related. These are, firstly, dilated Virchow–Robin spaces (VR), defined as small hypodense or cystic, non-enhancing, lesions with a diameter of 2 mm–3 mm on axial slices. Secondly, gelatinous pseudocysts, presenting as low-density and non-enhancing lesions, but because of accumulation of the yeast, bigger than VR spaces (>3 mm) and often located in the basal ganglia and periventricular areas. Cryptococcomas are a third specific finding and consist of masses also mostly located in the basal ganglia, with or without enhancement and surrounding oedema.^6,8,9,10,11^ In the setting of confirmed, active cryptococcal meningitis we would also define meningeal enhancement and hydrocephalus (dilated ventricular system with ballooning of third ventricle recess and temporal horns of the lateral ventricles, not attributed to atrophy) as cryptococcal-related radiological findings. Other non-specific findings included global cerebral atrophy and symmetrical confluent white matter changes in keeping with HIV encephalopathy.

## Results

Thirty-nine (44.8%) out of 87 HIV-infected patients admitted with cryptococcal meningitis between 2012 and 2013 underwent brain imaging in our institution. As we did not have an MRI scanner at the time, all imaging was restricted to CT imaging of the brain. We were able to retrieve and analyse 31 of the original scans. One of these scans was excluded because of poor quality (motion artefacts). The radiological findings are summarised in Table 1.

**TABLE 1:** Computerised tomography findings of 30 patients with confirmed cryptococcal meningitis.

Findings	*n* = 30	%
Normal imaging	4	13.3
CM-related findings	16	53.3
Dilated VR spaces	8	26.7
Pseudocyst	7	23.3
Hydrocephalus	5	16.7
Cryptococcoma	3	10
Soap bubble lesion	1	3.3
Meningeal enhancement	0	-
Other findings	-	-
Atrophy	17	56.7
Miscellaneous (incl. calcifications, white matter changes, enlarged cisterna magna)	7	23.3

*Source*: Authors’ own work

CM, cryptococcal meningoencephalitis; VR, Virchow–Robin.

**FIGURE 1:**
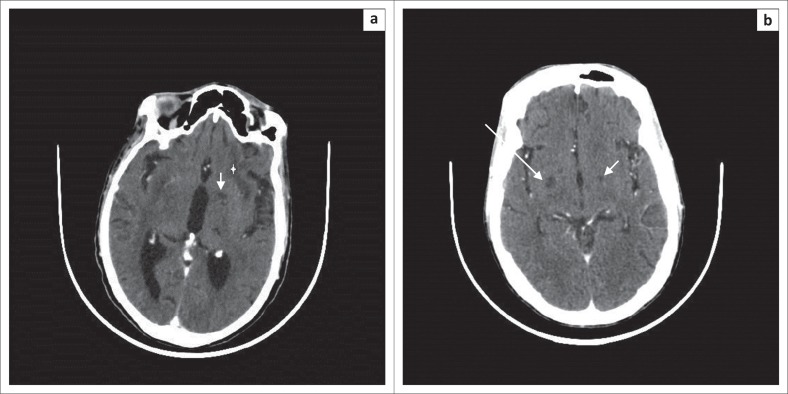
(a) Axial computerised tomography scan brain showing dilated Virchow–Robin (VR) spaces (arrow) as small hypodense non enhancing lesions in the left basal ganglia. Note also a larger hypodense lesion in the left external capsule consistent with a gelatinous pseudocyst, in addition, atrophy and mild hydrocephalus; (b) Axial brain scan with a pseudocyst in the right basal ganglia and a dilated VR on the left.

**FIGURE 2:**
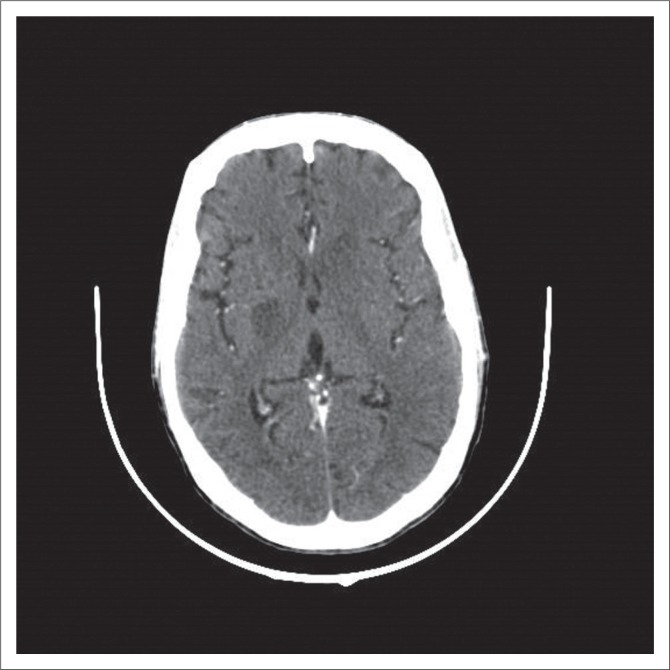
Axial contrasted computerised tomography scan with a non-enhancing cryptococcoma in the right basal ganglia.

Sixteen patient scans (53.3%) revealed neurocryptococcosis-related abnormalities. Dilated VR spaces (Figure 1a) were the most commonly found cryptococcosis-related pathological findings in our cohort, identified on 8 out of 30 scans (26.7%), followed by gelatinous pseudocysts (Figure 1b) in seven (23.3%), and hydrocephalus (Figure 1a) in five cases (16.7%). Cryptococcomas (Figure 2) were present in 10% of our patients, and we found one soap bubble lesion. The CT scan shown in Figure 1 illustrates some of the common radiological features in CM.

Completely normal CT scans were found in only four patients, another six patients had cerebral atrophy and four patients had miscellaneous findings (cerebral atrophy, white matter changes, calcifications and others), without cryptococcal-related pathology.

In Table 2, we compare our results with other studies on CT imaging in CM. Of note is the small percentage of normal scans seen in our study compared to other reports. The numbers of CT brains without pathology in our cohort seem more comparable with the reports of normal images obtained with MRI.

**TABLE 2:** Comparison of abnormal findings on computerised tomography brain imaging in HIV-associated neurocryptococcosis with other similar studies (in %).

Findings in %	Present study (*n* = 30)	Popovich et al.^9^ (*n* = 35)	Charlier et al.^6^ (*n* = 55)	Tien et al.^12^ (*n* = 29)	Moosa and Coovadia^13^ (*n* = 15)
Normal	13.3	43	47	31	80
Atrophy	56.7	34	6	45	Present^a^
Hydrocephalus	16.7	9	4	-	-
Dilated VRS	26.7	-	5	-	-
Pseudocysts	23.3	6	4	-	-
Cryptococcomas	10	6^b^	9	17	-
Diffuse oedema	-	3	4	-	-

*Source*: Authors’ own work

VRS, Virchow–Robin spaces.

a Number not published, only three abnormal scans (infarcts, incidental calcification, focal enhancement, one each);

b two lesions (one intraparenchymal, ring-enhancing and one intraventricular cryptococcoma).

Furthermore, five patients had communicating hydrocephalus on CT, representing 16.7% of the cohort. Compared with other reports, even with MRI, this was high. In Table 3, the details of the patients with hydrocephalus are listed.

**TABLE 3:** HIV patients with cryptococcal meningoencephalitis presenting with hydrocephalus on computerised tomography brain.

No	Gender	Age/a	Additional findings on CT	CD4 count (cells/µl)	ARVs	Presenting with
1	Female	42	Atrophy, enlarged cisterna magna	32	Naïve	Headache, meningism, vomiting
2	Male	32	-	67	Naïve	Headache, meningism, vomiting, focal deficit, seizures
3	Male	51	Atrophy	77	Naïve	Headache, meningism, focal deficit, seizures, confusion
4	Female	69	Atrophy	45	Naïve	Meningism, confusion, seizures, focal deficit,
5	Male	55	Dilated VRS, pseudocysts	61	Defaulter	Meningism, confusion

*Source*: Authors’ own work

ARVs, antiretroviral; CT, computerised tomography; VRS, Virchow–Robin spaces.

The mean CD4 count of the patients that were scanned was 44 cells/µL; and not greatly differing from the mean CD4 count in the whole cohort of CM patients of 52 cells/µL.

The overall mortality in the patients that underwent CT brain imaging was 33.3%, hence, very similar to the in-patient mortality in the total cohort of patients with cryptococcal meningitis (31%).^7^ In the group with cryptococcal-related changes on imaging, the mortality was higher (50%) and a small subgroup of patients with hydrocephalus showed a 100% mortality (*n* = 5). The patients with normal cerebral imaging and the group of patients that had cerebral atrophy and no other pathological findings, had lower mortalities at 25% and 16.7%, respectively.

Table 3 shows the details of the patients with hydrocephalus. The mean CD4 count of this subgroup was 69.8 cells/µL, therefore, slightly higher than the CD4 count in the entire group. However, none of these patients developed immune reconstitution inflammatory syndrome (IRIS).

Seventeen patients (56.7%) received concurrent treatment for TB while in hospital and on antifungal medication. This is similar to 56.3% of patients in the whole cohort of patients with CM. Only two of those had proven TB (one positive sputum culture and one positive on Sputum GenXpert). The majority of patients were either already on anti-mycobacterial treatment from other health care providers or restarted after they had defaulted TB treatment. Some were empirically started during their hospital stay for different reasons such as severe, non-resolving chest radiographs, pancytopenia or generalised lymphadenopathy. Other common co-infections were oral candidiasis (*n* = 8), onychomycosis and Herpes simplex infections.

## Discussion

Previously published data suggest a higher sensitivity of MRI compared with CT in the detection and evaluation of neurocryptococcosis-related radiological abnormalities in HIV patients. Charlier et al.^6^ report 92% abnormal MR scans at baseline contrasting with 53% abnormal CT scans in their retrospective analysis in 2008. In 17 of their patients who had dual imaging (CT and MRI), the MRI detected significantly more lesions (76% vs. 24%, *p* = 0.005) than CT. Dilated VR spaces were especially more frequently visualised by MRI than by CT. These findings support prior results and both modalities might well underestimate the extent of pathology when compared with post-mortem studies.^14^

The presence of abnormal brain imaging had a negative impact on survival, shown in a cohort study from France in 2007 where mortality at 3 months was significantly higher in patients with abnormal brain imaging.^15^

**FIGURE 3:**
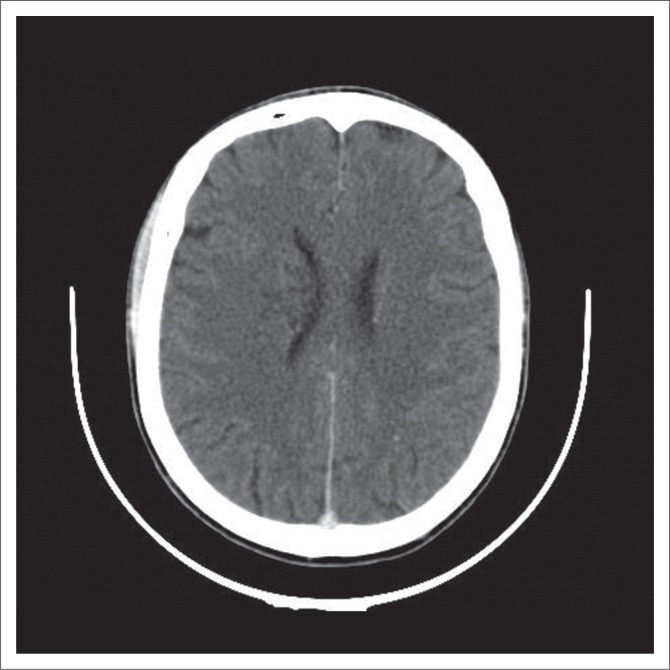
Classical soap bubble lesion in the right basal ganglia seen on this contrast-enhanced axial image.

We analysed in this retrospective spin-off study, the CT scans of 30 HIV patients with confirmed CM. For better comparability, we divided our positive findings into cryptococcal-related radiological pathology and ‘other’ abnormalities. Our study correlates with previous results in terms of qualitative findings. Consistent with When et al.,^16^ Tien et al.,^12^ Loyse et al.^5^ and others, we found dilated VR spaces the most common cryptococcosis-related radiological finding at almost 27%. In view of the assumed pathophysiology with the fungus spreading haematologically from the lungs to the brain, this is reasonable. Perivascular spaces are small areas with interstitial fluid created by blood vessels entering the meninges, usually only visualised when dilated, and they are the ‘first station’ for the fungus entering the CNS. VR spaces are predominantly found in the basal ganglia. When the fungal burden increases and mucous substances from the capsule of the organism are accumulating, dilated VR spaces expand to gelatinous pseudocysts, which by some authors are considered as specific for the disease.^17^ If clusters of these cysts develop, so called ‘soap bubble’ (Figure 3) lesions are found.^18^ Cryptococcomas (Figure 2) are intraparenchymal lesions representing a more chronic granulomatous reaction to the infection and, therefore, often not seen in the advanced stages of AIDS as there is little immune response.^19^ Radiologically, they are larger than the VR spaces and cysts and might be less hypodense. Contrast enhancement and surrounding oedema might be present in cryptococcomas in contrast to the latter. None of our patients had intraventricular cysts and only one had a cluster of cysts (soap bubbles). We also did not have any scan detecting meningeal contrast enhancement, which is also regarded as a feature of active disease. This is consistent with previous reports,^8^ and we believe it mirrors the fact of severe immunosuppression in our cohort with little inflammatory response and also reflects that there were no cases of IRIS amongst our patients.

Compared with other studies, we found a low number of completely normal scans at 13.3% (see Table 1). By adding the cases with ‘just’ atrophy (*n* = 6) we arrive at a percentage of 33%, which is still low. One possible explanation is the better resolution of our current scanner compared to the scanners used in the cited studies (Table 2), our data being from 2012 to 2013 and the studies published between 1997 and 2008. The data from the study by Moosa and Coovadia,^13^ showed the highest percentage of normal scans, were collected between 1991 and 1994, placing our scans at an approximately 20 year time- and technical advance.

The in-patient mortality in the patient group with CTs was not different than the mortality of all patients seen over the 2-year period with approximately one third of the patients dying in both groups. The groups did not differ significantly regarding CD4 counts or disease severity. Looking at patients with cryptococcal-related abnormalities on imaging, we found higher in-patient mortality in this group (50%) compared to the whole group, confirming the above mentioned results.^15^

Furthermore, we found a relatively high number of CT scans showing hydrocephalus, with or without other pathology (16.7%) and, additionally, all patients who had hydrocephalus died during their hospital stay, resulting in a 100% mortality rate of this subgroup of five patients. While few authors regard hydrocephalus as a non-specific but common feature of cryptococcal meningitis,^10^ others report cases with hydrocephalus less often (compared with results in Table 2). Even in MRI studies the prevalence of hydrocephalus was low (e.g. 2% in Loyse et al.,^5^ Charlier et al.^6^). There is sound data that raised intracranial pressure correlates with higher mortality and international and national guidelines support aggressive management if the baseline opening CSF pressure is raised.^20^ Our own data showed an increased mortality in those patients that were admitted with features consistent with high CSF pressures^7^ and the five patients with communicating hydrocephalus are probably part of this subgroup with poor prognosis. Unfortunately, as this was a retrospective study, opening pressures were mostly not documented and, therefore, we cannot compare and correlate the pressures with the imaging. One patient had a normal opening pressure documented and one an increased pressure, but in the remaining three cases the data were incomplete. Of note is that in none of the cases was the degree of the hydrocephalus notably extensive on imaging (Figure 1), to suggest the necessity for shunt surgery. Neither was there a non-communicating hydrocephalus implicating a contraindication for the procedure of a lumbar puncture.

There are obvious limitations to the present study. It is not entirely clear why certain patients underwent imaging and others did not and how this might have influenced our results. Although most clinicians decide to have a patient scanned in the presence of a reduced level of consciousness, signs of raised intracranial pressure or focal neurological deficit and seizures, not all patients with these features were scanned. There was also no guideline in our hospital, suggesting that all patients with confirmed neurocryptococcosis should have a CT scan of their brain. Often, it depended on the personal judgement by the admitting physician, the current burden of work in the radiology department or even trivial factors like the availability of a porter to get the patient in to the scanner. As the nature of this study is retrospective, the available data are not complete, and because of the small numbers we went, without statistical analysis, for a descriptive study.

## Conclusion

The neuroradiological findings in our patients with HIV-associated CM are qualitatively similar to previously reported findings. The most common crypto-related abnormality was the presence of dilated VR spaces. Our yield in detecting abnormalities on imaging was higher than in previous studies. This finding suggests that modern CT imaging represents a good alternative to MRI in resource-limited settings. The presence of cryptococcal-related abnormalities on CT brain is indicative of a higher mortality. The subgroup of patients with communicating hydrocephalus was associated with 100% mortality in our cohort, emphasising the importance of appropriate management of raised intracranial pressure.
